# Treatment expectation among patients with diabetes and hypertension in a tertiary hospital, Ekiti State, Southwest Nigeria: a cross-sectional study

**DOI:** 10.11604/pamj.2023.45.143.38577

**Published:** 2023-07-26

**Authors:** Abigael Adeoye, Oluwaseyi Akpor, Stephen Oguntola, Oghenerobor Akpor

**Affiliations:** 1 Department of Nursing Science, Afe Babalola University, Ado-Ekiti, Ekiti State, Nigeria,; 2 Renal Unit, Afe Babalola University Multisystem Hospital, Ado-Ekiti, Ekiti State, Nigeria,; 3 Department of Biological Sciences, Afe Babalola University, Ado-Ekiti, Ekiti State, Nigeria

**Keywords:** Treatment expectation, diabetes, hypertension, perception, satisfaction

## Abstract

**Introduction:**

patients' satisfaction is an important aspect in determining the quality of health care since it reveals how staff are progressing toward the patients' objectives.

Objective: the study determined the treatment perception and expectations among diabetes and hypertensive patients in a teaching hospital in Ekiti State, Nigeria.

**Methods:**

the study employed a cross-sectional design approach with a sample size of 200 participants. Descriptive analyses were used to answer the research questions while inferential statistics were used to test hypotheses at a significant level of p < 0.05.

**Results:**

the findings revealed that 50% (n=196) and 57.1% (n=196) of the patients with diabetes and hypertension were above 60 years with mean age and standard deviation being 3.36 ± 0.72, while 63.3% (n=196) and 64.3% (n=196) of the patients with diabetes and hypertension were females. Overall, the results revealed a significant difference between the domains of participants´ expectations and perceptions, with the expectations domains being significantly higher for both diabetes and hypertensive patients with mean score and standard deviation being (-5.14 (±1.62) and -4.55 (±1.83)) respectively. However, the difference in the gap scores between the participants with diabetes and those with hypertension across the domains of patients´ expectations and perceptions was significant. Furthermore, apart from the tangible domain 19.76 (±0.87) (p < 0.05), the findings from the study showed that participants with diabetes had significantly higher expectation scores, when compared with those with hypertension in all domains. Additionally, the participants with diabetes showed the highest level of perception in the tangible 15.75 (±1.43) and empathy 20.50 (±1.20) domains while those with hypertension showed the highest level of perception in the reliability, responsiveness, and assurance domains (21.66 (±2.45), 16.58 (±1.38) and 21.43 (±2.03) p < 0.001 respectively).

**Conclusion:**

efforts should be intensified by nurses and all other stakeholders to exceed patients´ expectations by continually improving the quality of health care and services offered to patients.

## Introduction

Patients’ satisfaction is an important aspect in determining the quality of health care since it reveals how the staff is progressing toward the patients' objectives. It is a significant factor in the patient's expectations [1]. Thus, a determination of treatment satisfaction among chronically ill patients could be a first step toward determining an individual´s ability to follow healthy behaviors [2]. Furthermore, it is the patient's assessment of how well their expectations were satisfied by the care supplied, or an appraisal, that serves as the foundation for the patient's expectations being met [3].

Aydin [4] identified eight aspects that influence patient satisfaction with the healthcare system: the art of care, technical quality of treatment, accessibility and convenience, finances, physical environment, availability of resources and personnel, continuity, and efficacy with the outcome of care.

Diabetes mellitus (DM) is a chronic metabolic disease that is characterized by constant high blood glucose levels that are connected to severe body organs damage such as the heart, blood vessels, eyes, kidneys, and nerves over time [5,6]. Globally, the rate of diabetes is increasing and it has become a public health challenge, with a higher burden among developing countries such as Nigeria [7-9]. Preventive care is less common in low- and middle-income countries with a greater burden in countries with fewer health care resources [10].

Hypertension is defined by the World Health Organization [6] as persistently elevated systolic blood pressure greater than 140 mmHg and diastolic pressure greater than 90 mmHg. Although hypertension is preventable, it is still a major public health problem globally, its prevalence has been increasingly rising [11,12]. Hypertension ranks first among the preventable causes of death worldwide and the number of patients with hypertension continues to increase in developing countries every year [11,13]. The prevalence also varies by ethnicity, with African Americans having the highest prevalence [14].

Patient satisfaction is linked to better adherence to treatment processes and recognized prevention, as well as improved health outcomes [3]. Hypertensive and diabetes patients must be satisfied with their treatment to enhance adherence to the treatment regimen thus, improving their quality of life and minimizing complications. Consequently, there is a need to look into the current discrepancies between patients' expectations of healthcare services and their perceptions of such services after they have been delivered. Therefore, the study investigated treatment expectations among patients with diabetes and hypertension in a tertiary hospital in Ado Ekiti, Ekiti State.

## Methods

**Study design and setting:** the study adopted a descriptive research design with a quantitative strategy. The study setting was Ekiti State University Teaching Hospital, Ado Ekiti, Ekiti State, Nigeria. The tertiary hospital was commissioned on March 9, 1971, to serve the rising population of Ado-Ekiti. The hospital's departments include Obstetrics and Gynecology, as well as Pediatrics. Medicine, Family Medicine, Surgery, Accident and Emergency, Psychiatry, Ear, Nose, and Throat, and other medical specialties are available. On Wednesday and Tuesday of each week, the hospital hosts an outpatient clinic, which provides follow-up care for patients living with diabetes and hypertension respectively.

**Study population:** the target population is the entire population, or group, that a researcher is interested in researching and analyzing [15]. The target population for the study was diabetes and hypertensive patients receiving follow-up care at the teaching hospital that met the eligibility criteria. The inclusion criteria for this study were diabetes and hypertensive patients who were at least 18 years old and willingly volunteered to participate in the study. Patients with diabetes and hypertension who were seriously ill and those not willing to participate in the study were excluded.

Purposive and convenience sampling techniques were used for the study. The purposive sampling method was used to select diabetes and hypertensive patients receiving follow-up care at the hospital. Patients who met the inclusion criteria were conveniently recruited during their clinic follow-up care which is on Tuesdays for the cardiology clinic and Wednesdays for the endocrinology clinic. According to the hospital records of December 2021, approximately 30-35 and 40-45 patients attended the endocrinology and cardiology clinics weekly, respectively. A sample size of 187 comprising of diabetes and the hypertensive patients who attends follow-up care in the teaching hospital formed the sample for this study. However, 10% of non-response was anticipated hence a total of 200 respondents was therefore used for the study.

**Data collection:** an adapted questionnaire with biophysical measurements were used for data collection. The questionnaire was adapted from Katuti [16]. The questionnaire consisted of three sections. Section A derived information on the demographical status of the participants while section B included questions on clinical characteristics while section C assessed the level of patients' satisfaction with health services delivery and factors influencing patients' satisfaction with the quality of health care services. Health system factors were scored as high, moderate, and low. High factors (3) were calculated as scores from 1 - 18, moderate factors (2) from 19 - 26, while low factors (3) were scores from 27 - 38.

**Definitions:** the biophysical measurements conducted were blood pressure, weight, height, and body mass index (BMI). Body mass index (BMI) was calculated as: BMI = weight (kg)/height (m)^2^. Categorization of BMI was as classified by WHO (2021): underweight (<18.5 kg/m^2^), ideal weight (18.5 - 24.9 kg/m^2^), overweight (25.0 - 29.9 kg/m^2^), and obese (≥30 kg/m^2^).

Participants´ heights and weights were measured with a medical giraffe height measuring stadiometer (Model HMS PL) and analog flat weighing scale, respectively. Measurements were taken to the nearest 0.1 m and 0.1 kg, for height and weight, respectively. Blood pressure (BP) was measured using a digital sphygmomanometer (Omron M2 ECO) while the participant was in a sitting position after a rest of about 5-10 min was ensured. The BP recordings were recorded to the nearest whole number.

**Statistical analysis:** data were presented in the form of descriptive statistics and inferential statistics. Participants´ responses from the questionnaire were summarized frequencies and proportions while clinical characteristics of respondents were calculated in the form of means and standard deviations. The One-Way Analysis of variance (ANOVA) was used to calculate the significance of means while the Chi-Square analysis was used to test for relationships. All inferential statistics were carried out at a 95% confidence interval. All data were analyzed using the Statistical Package for Social Statistics (SPSS), version 23.0.

**Ethical consideration:** before the commencement of the study, approval was obtained from the Ethics Committee of the Ekiti State University Teaching with protocol number EKSUTH/A67/2022/01/001. In addition, the participants´ autonomy was respected, as the study respondents were adequately informed on the process, purposes, and objectives of the study. Willing respondents were used for the study without coercing anyone to take part in the research. The physical, mental, and social well-being of participants was safeguarded and the study did not cause any emotional/mental or social harm to the respondents. Furthermore, confidentiality was ensured by handling all information cautiously during data collection and data processing. For all categories of participants informed consent form was filled, and duly signed.

## Results

**Socio-demographic and biophysical profile of the participants:** the socio-demographic profile of the participants revealed that the majority (50% (n=196) for those with diabetes and 57.1% (n=196) for those with hypertension) were above 60 years of age. Over 60% (n=196) of the participants were females. With respect to duration of diagnosis of ailment, the majority of the participants with diabetes (38.8% (n=196)) have had the disease for more than 10 years while the majority of participants with hypertension (35.7% (n=196)) have had the disease for between 3-4 years (Table 1).

**Table 1:** demographic and clinical characteristics of the participants (N=196)

Characteristics		Diabetes		Hypertension	
		**Frequency**	**Percentage**	**Frequency**	**Percentage**
**Age (years)**	25-39	14	14.3	10	10.2
	40-60	35	35.7	32	32.7
	Above 60	49	50.0	56	57.1
**Gender**	Male	36	36.7	35	35.7
	Female	62	63.3	63	64.3
**Level of education**	No formal education	7	7.1	3	3.1
	Primary	11	11.2	26	26.5
	Secondary	42	42.9	14	14.3
	Tertiary	38	38.8	55	56.1
**Employment status**	Employed	12	12.2	22	22.4
	Business/private	50	51.0	34	34.7
	Not employed	9	9.2	11	11.2
	Retired	27	27.6	31	31.6
	Above 100,000	19	19.4	14	14.3
**Religion**	Islam	12	12.2	17	17.3
	Christianity	86	87.8	81	82.7
**Duration of diabetes (years)**	1-2	9	9.2	3	3.1
	3-4	7	7.1	6	6.1
	5-6	34	34.7	4	4.1
	7-9	10	10.2	4	4.1
	>10	38	38.8	-	
	No diabetes	-	-	81	82.7
**Duration of hypertension (years)**	1-2	7	7.1	3	3.1
	3-4	10	10.2	35	35.7
	5-6	3	3.1	25	25.5
	7-9	16	16.3	16	16.3
	>10	-	-	19	19.4
	No hypertension	62	63.3	-	-
**Biophysical characteristics**		**Mean**	**Range**	**Minimum**	**Maximum**
**Participants with diabetes**					
Weight (Kg)		71.80 ± 10.83	56	54	110
Height (m)		1.56 ± 0.04	0.30	1.40	1.70
BMI score (kg/m^2^)		29.64 ± 4.39	24.40	20.80	45.20
Systolic blood pressure (mmHg)		137.20 ± 17.36	70	110	180
Diastolic blood pressure (mmHg)		83.22 ± 7.92	40	60	110
**Participants with hypertension**					
Weight (Kg)		77.76 ± 15.28	63	47	110
Height (m)		1.50 ± 0.74	0.62	1.10	1.72
BMI score (kg/m^2^)		30.25 ± 5.49	20.30	18.70	39.00
Systolic blood pressure (mmHg)		142.49 ± 20.12	73	107.00	180.00
Diastolic blood pressure (mmHg)		87.50 ± 12.68	49	63	112

Biophysical characteristics of the participants revealed mean body mass index (BMI) of 29.64 kg/m^2^ and 30.25 kg/m^2^ for participants with diabetes and hypertension, respectively. In addition, the mean systolic and diastolic blood pressures of the participants were observed at 137.20 and 83.22 mmHg and 142.49 and 87.50 mmHg, for participants with diabetes and hypertension, respectively (Table 1).

**Participants´ expectation and perception of treatment:** generally, participants´ expectation scores were significantly higher than scores for perception (p < 0.05). This observation was irrespective of the disease conditions or the respective dimensions. Among the participants with diabetes, the lowest and highest mean gap scores of -2.90 and -5.14 were observed in the tangibles and reliability dimensions, respectively. In the case of participants with hypertension, the lowest and highest mean gap scores of -2.85 and -4.55 were observed in the reliability and empathy dimensions, respectively (Table 2).

**Table 2:** participants’ expectation and perception scores across the five dimensions of treatment

Dimension		Mean	Gap	p-value
**Participants with patients with diabetes**				
Tangibles	Expectation	18.64 ± 4.82	-2.90	p<0.001
	Perception	15.74 ± 1.43		
Reliability	Expectation	25.00 ± 0.00	-5.14	p<0.001
	Perception	19.86 ± 1.62		
Responsiveness	Expectation	20.00 ± 0.00	-4.15	p<0.001
	Perception	15.85 ± 1.27		
Assurance	Expectation	25.00 ± 0.00	-4.81	p<0.001
	Perception	20.19 ± 1.93		
Empathy	Expectation	25.00 ± 0.00	-4.50	p<0.001
	Perception	20.50 ± 1.10		
**Participants with hypertension**				
Tangibles	Expectation	19.76 ± 0.87	-4.32	p<0.001
	Perception	15.44 ± 2.64		
Reliability	Expectation	24.51 ± 1.38	-2.85	p<0.001
	Perception	21.66 ± 2.45		
Responsiveness	Expectation	19.46 ± 2.61	-2.88	p<0.001
	Perception	16.58 ± 1.38		
Assurance	Expectation	24.96(±0.20)	-3.53	p<0.001
	Perception	21.43 ± 2.03		
Empathy	Expectation	24.94 ± 0.35	-4.55	p<0.001
	Perception	20.40 ± 41.81		

Apart from the tangible dimension, the expectations scores of participants were observed to be higher for participants with diabetes across the different dimensions. Significant differences in expectations scores between the two categories of participants were observed to be significant different (p < 0.05) for empathy, reliability, responsiveness, and assurance dimensions (Table 3).

**Table 3:** comparison of expectation and perception scores between the two categories of participants

Healthcare dimension	Participants’ type	Mean	p-value
**Expectation**			
Tangible	Hypertension	19.76 ± 0.87	0.026
	Patients with diabetes	18.64 ± 4.83	
Reliability	Hypertensive	24.51 ± 1.38	0.001
	Patients with diabetes	25.00 ± 0.00	
Responsiveness	Hypertensive	19.46 ± 2.61	0.042
	Patients with diabetes	20.00 ± 0.00	
Assurance	Hypertensive	24.96 ± 0.20	0.044
	Patients with diabetes	25.00 ± 0.00	
Empathy	Hypertensive	24.94 ± 0.35	0.082
	Patients with diabetes	25.00 ± 0.00	
**Perception**			
Tangible	Hypertensive	15.44 ± 2.64	0.314
	Patients with diabetes	15.75 ± 1.43	
Reliability	Hypertensive	21.66 ± 2.45	p<0.001
	Patients with diabetes	19.86 ± 1.62	
Responsiveness	Hypertensive	16.58 ± 1.38	p<0.001
	Patients with diabetes	15.85 ± 1.27	
Assurance	Hypertensive	21.43 ± 2.03	p<0.001
	Patients with diabetes	20.19 ± 1.93	
Empathy	Hypertensive	20.40 ± 1.81	0.633
	Patients with diabetes	20.50 ± 1.20	

In the case of perception, higher scores of 15.75 ± 1.43 and 20.50 ± 1.20 were observed for participants with diabetes in the tangible and empathy dimensions, respectively while for reliability, responsiveness, and assurance dimensions, higher scores of 21.66 ± 2.45, 16.58 ± 1.38 and 21.43 ± 2.03 were observed for participants with hypertension, respectively. Generally, perception scores between participants with diabetes and those with hypertension were observed to be significantly (p < 0.05) different in the reliability, responsiveness, and assurance categories (Table 3).

## Discussion

The study determined the treatment perception and expectations among diabetes and hypertensive patients in a teaching hospital in Ekiti State, Nigeria. Generally, participants´ expectation scores were significantly higher than their perception scores. Apart from the tangible dimension, the expectations scores of participants were observed to be higher for participants with diabetes across the different dimensions. In the case of perception, higher scores were observed for participants with diabetes in the tangible and empathy dimensions and while reliability, responsiveness, and assurance scores were higher for participants with hypertension.

The socio-demographic characteristics of the participants revealed that with respect to the age distribution of the participants, more than half of the diabetes and hypertensive participants were above 60 years, this result is contrary to the findings of a similar study in Ebonyi State, Nigeria by Umoke *et al*. [1] where the majority of the participants ages ranged between 18 and 39 years. Also, almost two-thirds of diabetes and hypertensive participants respectively were female, this is similar to the findings obtained by Umoke *et al*. [1] with 60.6% (n=196) of the study population being female while its converse to the findings by Fan *et al*. [17] who derived an equal proportion of male and female in their study in China.

The significance of quality in health care cannot be underrated as the quality of a healthcare service delivered is whatever the patient perceives (patients´ experiences of services available in the hospital) which may differ from the quality of the service actually expected (what should be available in the hospital) for all domains: tangibles, reliability, responsiveness, assurance, and empathy. An attempt to determine whether there is a pact between participants´ perceptions and expectations of health care services using the service and quality (SERVQUAL) tool was made by the study. Assessing the gap between the patient´s expectations and perceptions of health care services will provide hospitals with insights on how to improve their quality of care and enhance practices to facilitate patient satisfaction.

Overall, the results revealed a significant difference between the domains of participants´ expectations, and perceptions, with the expectations domains being significantly higher for both diabetes and hypertensive patients respectively. This is in agreement with a study conducted by A´aqoulah *et al*. [18] in two hospitals in Jordan and in another study conducted in the General Out Patients Department of a Teaching Hospital in Jordan. The finding was also in alignment with a study conducted in five private hospitals in a province in Tehran, Iran by Nazem *et al*. [19]. A study conducted in South Eastern part of Nigeria by Umoke *et al*. [1] also supported this finding. This depicted a level of dissatisfaction with the quality of healthcare services received by the patients and a need for a global improvement in healthcare systems.

However, there were differences in the gap scores between the diabetes and hypertensive participants across the domains of patients´ expectations and perceptions. For the diabetes participants, they have the lowest gap score in the tangible domain and the highest gap score in the reliability domain while the hypertensive participants have the lowest gap score in the reliability domain and the highest gap score in the empathy domain. This is to emphasize that the expectations and perceptions of each group differ as the diabetes participants longed that they receive reliable health care services which include: services and procedures performed without errors and mistakes, services should be provided within the time promised, patients records should be without error while the hypertensive participants desired empathetic care which includes: hospitals to operate at times suitable for patients, health care providers to listen to patients attentively, staffs should understand the specific needs of each patients. This is in tandem with the study conducted by Umoke *et al*. [1] where they established the importance of each domain to patient satisfaction. Contrastingly, Nazem *et al*. [19], in their study identified the responsiveness domain which includes: staff should be willing to help the patients, waiting time for admission and daily services should be short has the least gap score among the study population. Also, according to Teshnizi *et al*. [20] a negative gap means that patients´ expectations for quality healthcare services was not met which also advocates that healthcare directors and policymakers should pay more attention to patients´ rights and be more receptive to perceived shortcomings.

Furthermore, the findings from the study showed that the diabetes participants have the highest level of expectations in all domains except the tangible domain with significant differences in the expectations scores between the two groups. This is to say that they are not really concerned about the infrastructural facilities of the health facility as much as they are concerned about the skills, expertise, and experiences of the health care providers, and the degree of the health providers´ willingness to help and provide empathetic care. This is similar to one of the studies reviewed in a meta-analysis conducted by Teshnizi *et al*. [20].

Also, the diabetes participants have the highest level of perception in the tangible and empathy domains respectively, this is similar to the findings by Teshnizi *et al*. [20] this implied that the healthcare providers are seemingly more intentional about tangibility and empathy, and take measures to ensure patients´ expectations in these domains are met. In contrast, participants with hypertension had a higher level of perception in the reliability, responsiveness, and assurance domains respectively. This posited that of the three dimensions of service quality, the participants´ seemed to be most satisfied with access and expertise, followed by the human aspect and less satisfaction from the physical environment and infrastructure dimensions. This is in agreement with the meta-analysis study conducted by Teshnizi *et al*. [20].

The findings from the study thus required that healthcare providers should be trained properly in effective skills to enhance their interpersonal and communication skills with patients. Furthermore, regular in-service training on strategic healthcare provider-patient relationships and strategies to reduce patient waiting time, and advocating with hospital management on the provision of structures and setting that will promote patients´ satisfaction.

Despite the fact that the study's goals were met, it may still have some limitations. The scope of this study was limited to patients with diabetes and hypertension receiving follow-up care at the teaching hospital in Ekiti State. Thus, the findings cannot be generalized to a larger context. Also, a subjective self-reported assessment tool was used. Notwithstanding, the evaluation of patient satisfaction from the view of patients with chronic health conditions with frequent hospital visit and follow-up can serve as the baseline for further research, particularly on the relationship between patients' satisfaction and health outcomes in areas of treatment compliance and quality of life.

## Conclusion

The study revealed a significant difference between all the domains of participants´ expectations and perceptions explored which showed that patients were not satisfied with the quality of healthcare services they received and their expectations were not met. Hence, the need for, proper identification of individual patients´ expectations from healthcare service providers and improvement in the quality of care and services rendered to patients which will ultimately facilitate patient satisfaction and promote positive health outcomes.

### What is known about this topic

- *Patient satisfaction is linked to better adherence to treatment processes and recognized prevention, as well as improved health outcomes;*
- *Patients´ satisfaction is a crucial component in determining the quality of healthcare services provided;*
- *It offers insight into how well healthcare professionals are meeting patients' needs*.

### What this study adds

- *The study revealed significant differences in the gap scores between the patients with diabetes and hypertension across the domains of patients´ expectations and perceptions;*
- *Variation in scores across the studied domains by each studied group of patients revealed that individualized care is paramount in promoting patients´ satisfaction with healthcare services;*
- *Overall, the data generated can serve as the baseline for further research, particularly on the relationship between patients' satisfaction and health outcomes*.
